# Nestle’s Milk Food

**Published:** 1882-02-20

**Authors:** 


SEE advertisement of Nestle's Milk Food. This article comes highly recommended and will be found well adapted to infants and invalids as an easily digested article of food.

